# Predicting Factors of Cognitive Flexibility in Chinese–English Bilinguals: Insights from Mouse Tracking Task Switching

**DOI:** 10.3390/bs15111481

**Published:** 2025-10-30

**Authors:** Wenting Ye, Mengyan Zhu, Ting Li, Jiang Qiu

**Affiliations:** 1 Faculty of Psychology, Southwest University (SWU), Chongqing 400715, China; wy14940@swu.edu.cn; 2 Key Laboratory of Cognition and Personality (SWU), Ministry of Education, Chongqing 400715, China; 3 School of Psychological Science, University of Bristol, Bristol BS8 1QU, UK; mengyan.zhu@bristol.ac.uk; 4 Faculty of Social Sciences, Chongqing University, Chongqing 400044, China; 15035030872@163.com

**Keywords:** bilingual, task switching, switch cost, mix cost, mouse tracking

## Abstract

This study investigated factors predicting cognitive flexibility in Chinese–English bilinguals, with a comprehensive focus on demographic and language-related variables. Cognitive flexibility was assessed using reaction times (RTs) and maximum absolute deviation (MAD) in a mouse-tracking nonverbal task-switching paradigm, capturing both mix and switch costs. Regression analyses revealed that bilingual experience explained a larger proportion of variance in mix costs than in switch costs, with stronger effects for MAD than RTs. Higher composite factor scores (CFS) were positively associated with mix costs, whereas balanced language use across life stages, activities, and interlocutors predicted smaller mix costs, suggesting a move to multi-dimensional, experience-based approaches. In contrast, switch costs were largely unrelated to CFS, but balanced language use across situational contexts, which predicted reduced switch costs in MAD, indicating enhanced reactive control. Moreover, bilingual experiences in the home environment appeared to be positively associated with cognitive flexibility. These findings highlight the multidimensional nature of bilingual experience and underscore the value of movement trajectory measures in capturing subtle effects on sustained and transient cognitive control.

## 1. Introduction

Bilingualism has long been proposed to influence domain-general cognitive control, including the ability to flexibly switch between tasks, often referred to as cognitive flexibility (Bialystok, 2017; Grundy et al., 2017; Prior & MacWhinney, 2010). A large body of task-switching research has examined cognitive flexibility using laboratory tasks in which participants classify stimuli along one of two possible dimensions. For example, in a nonverbal colour-shape task switching paradigm (e.g., Hartanto & Yang, 2016; Prior & MacWhinney, 2010), bivalent stimulus (e.g., a red or blue triangle or circle) are presented on the screen, and during each trial, a cue indicated whether participants should respond to the colour (red vs. blue) or the shape (triangle vs. circle) of the stimulus. In *pure* blocks, participants judged only one dimension of the stimulus (either colour or shape). In *mixed* blocks, both sets of cues were presented, and participants had to judge the relevant dimension based on the cue. This design yields two widely used performance indices in the task-switching literature. *Switch cost*, defined as slower responses and/or increased errors on trials in mixed blocks where the required task differs from the previous trial, compared to trials where the task repeats (Rogers & Monsell, 1995). This measure reflects transient, reactive control processes involved in reconfiguring the task set and overcoming interference from the competing task (Kiesel et al., 2010; Monsell, 2003; Vandierendonck et al., 2010; Ye & Damian, 2022); and *mix cost*, refers to the slowing and/or increase in errors in mix blocks compared with pure blocks (Strobach et al., 2012), indexing sustained, proactive control processes required to maintain and monitor multiple task sets simultaneously (Braver et al., 2003; Los, 1996). Prior and MacWhinney (2010) compared young adult bilinguals, who reported regular daily use of two languages since childhood, with monolingual peers on a non-linguistic colour-shape task switching paradigm. They found that bilinguals exhibited significantly smaller switch costs than monolinguals, indicating more efficient control in shifting between tasks, although no differences emerged in mix costs.

However, other studies report limited or no such influence of bilingualism on cognitive flexibility (Lehtonen et al., 2018; Paap et al., 2015). One possible reason for these inconsistent findings is the challenge of precisely defining and measuring “bilingual experience” (Anderson et al., 2018; de Bruin, 2019; Luk & Bialystok, 2013; Surrain & Luk, 2019). While numerous studies have investigated the relationship between bilingualism and cognitive flexibility using non-verbal paradigms (de Bruin, 2019; Kroll & Bialystok, 2013; Laine & Lehtonen, 2018), yet the operationalisation of bilingual experience has varied greatly across studies. Some researchers have adopted a broad categorical contrast between bilinguals and monolinguals (e.g., Prior & MacWhinney, 2010; Ward & Awani, 2024), others have attempted to capture individual variation in bilingual experience by collecting self-reported measures, such as age of acquisition, daily language use, and proficiency through standardized questionnaires (e.g., Khodos & Moskovsky, 2020; Khodos et al., 2021).

To address this issue, recent studies have sought to refine the measurement of bilingual experience by distinguishing its subcomponents, such as language use patterns, language switching pattern and interactional contexts. For example, Hartanto and Yang (2016) classified bilingual participants into single-language (i.e., primarily used one language in each context, with relatively little mixing) and dual-language (i.e., regularly used both languages across multiple contexts and engaged in frequent code-switching) contexts based on self-reported language use and everyday switching habits, showing that bilinguals from dual-language contexts exhibited smaller switch costs than those from single-language contexts. To provide more systematic assessment, Anderson et al. (2018) developed the Language and Social Background Questionnaire (LSBQ), which captures proficiency, age of acquisition, and language use across home and social domains, and has since been adopted in several task-switching studies. For example, Khodos and colleagues (Khodos & Moskovsky, 2020; Khodos et al., 2021) extended the LSBQ by incorporating variables such as interactional context (bilingual dual-language, separated-language, and monolingual contexts) and linguistic distance between languages (see more details in Khodos et al., 2021). In addition to the LSBQ, other widely used instruments include the Language History Questionnaire (LHQ; P. Li et al., 2014) and the Language Experience and Proficiency Questionnaire (LEAP-Q; Marian et al., 2007), both of which provide comprehensive measures of language background, proficiency, and use, and are also broadly applied in bilingualism research.

Similarly, Hartanto and Yang (2020) expanded upon their earlier work (Hartanto & Yang, 2016) by examining how distinct bilingual interactional contexts, namely dual-language contexts (i.e., where both languages are used within the same environment but typically with different interlocutors) and dense code-switching contexts (i.e., where elements of both languages are frequently mixed within a single conversation), predict individual differences in executive functions using a latent-variable approach based on self-reported language-use frequencies, compared with single-language contexts (i.e., when bilinguals use one language in one situation and the other language in a separate context). They found that bilinguals from dual-language contexts showed enhanced task-switching ability (as reflected in smaller switch and mix costs across paradigms including the colour-shape switching, magnitude-parity switching, and animacy-locomotion switching tasks). In contrast, those from dense code-switching contexts exhibited stronger inhibitory control (indicated by reduced interference effects in Arrow Flanker, Colour Flanker, and Eriksen Flanker tasks, as well as better working-memory updating) and maintenance (reflected in more accurate performance on Operation-Span, Rotation-Span, and Symmetry-Span tasks). These findings underscore that the cognitive effects of bilingualism depend not merely on language proficiency or usage frequency, but on the ecological demands of everyday language interaction patterns. The introduction of interactional context aligns with the Adaptive Control Hypothesis (Green & Abutalebi, 2013), in which it is proposed that the demands of different interactional contexts shape the recruitment and adaptation of domain-general control processes, and thus may play a role in cognitive flexibility.

Previous research has emphasized that bilinguals vary considerably in their interactional contexts (Hartanto & Yang, 2016, 2020; Khodos & Moskovsky, 2020; Khodos et al., 2021). While some individuals use their languages in clearly separated environments, others engage in extensive mixing and switching across multiple domains. It is true that much of the interest in linking bilingual experience to cognitive flexibility stems from the idea that frequent language switching may strengthen domain-general control, as formalized in the Adaptive Control Hypothesis (Green & Abutalebi, 2013). From this perspective, language switching recruits executive processes such as inhibition, reconfiguration, and conflict monitoring, which overlap with those engaged during non-verbal task switching (Bialystok, 2017; Grundy et al., 2017; Kroll & Bialystok, 2013; Lehtonen et al., 2018; Paap et al., 2015). Therefore, developing systematic measures of language-switching behaviour becomes essential for advancing our understanding of bilingualism in cognitive flexibility.

More recently, Ward and Awani (2024) conducted a close replication of Wiseheart et al. (2016) to re-examine whether bilingualism enhances task switching. They compared bilingual and monolingual young adults on a non-verbal cued task-switching paradigm involving colour and shape judgments. Instead of administering a comprehensive language background questionnaire such as the LSBQ (Anderson et al., 2018), they collected a few brief self-report measures assessing how frequently bilingual participants switched languages with family, friends, and on social media. These switching-frequency ratings were analyzed only within the bilingual group to explore whether everyday switching behaviours predicted task performance. The correlations between switching frequency and both mix costs and switch costs were nonsignificant, potentially suggesting that these coarse measures might not capture variability in bilingual language experience. Nevertheless, the main analysis revealed that bilinguals showed reduced mix costs compared with monolinguals, consistent with a domain-general advantage in cognitive flexibility (Bialystok, 2017; Prior & MacWhinney, 2010; Wiseheart et al., 2016).

To better account for language switching, Gullifer and Titone (2020) introduced language entropy as a principled, quantitative index that captures the diversity and balance of bilingual language use across social domains. By applying Shannon’s entropy formula (Shannon, 1948) to self-reported proportions of L1 and L2 use, they established a standardized, continuous measure that avoids the limitations of simple frequency counts. Entropy values range from low to high, with low values indicating dominance of one language in a given context and high values reflecting a more balanced distribution of both languages (Gullifer & Titone, 2020). Building on this framework, several recent studies have applied language entropy to bilingual task switching. For instance, X. Li et al. (2021) showed that higher entropy was associated with more specialized brain network organization and reduced variability in executive control tasks, including non-verbal task switching. Similarly, van den Berg et al. (2022) reported that higher entropy outside of university contexts predicted smaller mix costs and reduced switching-related pupil dilation in a colour-shape task. Together, these findings suggest that diverse and balanced language use, as quantified by entropy, may enhance the efficiency of cognitive control mechanisms underlying task switching.

Overall, despite growing recognition of multiple key dimensions underlying bilingual experience (e.g., proficiency, language use and language switching across context), limited work has attempted to integrate them within a unified and comprehensive framework. The present study thus aims to systematically capture bilingual experience within a single framework by combining standardized measures of proficiency and language use with language entropy, which indexes switching behaviours (see The Present Study and Method sections for details). We applied this framework to a nonverbal task-switching paradigm, examining switching and mix costs as behavioural markers of control. In addition to traditional reaction-time measures, we incorporated mouse tracking to capture a continuous record of task-set competition during decision making (Freeman & Ambady, 2010; Hehman et al., 2015). Specifically, we used the maximum absolute deviation (MAD), defined as the largest perpendicular deviation of the mouse trajectory from an ideal straight path toward the target. Previous studies have shown that experimental manipulations can reliably emerge in mouse movement, such as in conflict tasks and in task switching paradigm (e.g., Ye & Damian, 2022, 2023). Compared with key-press measures, which capture only discrete outputs, mouse tracking could provide richer and more dynamic information about how decisions unfold over time. While RT reflects both preparation and decision processes, MAD more directly captures the real-time competition. In the context of task switching, MAD reflects the degree of competition between concurrently activated task sets: when two task sets are coactivated, the mouse trajectory is “attracted” toward the competing option, resulting in greater curvature. This measure also enables us to examine how bilingual experience modulates the balance between reactive control (indexed by switch costs) and proactive control (indexed by mix costs) during the continuous and ongoing cognition, thereby offering a more comprehensive account of how bilingualism shapes cognitive control dynamics.

### The Present Study

In summary, the present study aims to use more comprehensive measures to clarify the role of bilingualism in cognitive flexibility. To achieve this, we employed a systematically standardized approach that integrates multiple dimensions of bilingual experience collected through established instruments. Specifically, we administered the LSBQ (Anderson et al., 2018) together with the LEAP-Q (Marian et al., 2007) and the LHQ 2.0 (P. Li et al., 2014). These tools allowed us to gather detailed demographic information (e.g., age, gender, education, and socioeconomic status), as well as linguistic factors including but not limited to age of bilingualism, language proficiency, language use across domains (situations, interlocutors, activities, etc.), and switching habits. To capture language-switching behaviours, we further applied language entropy measures across distinct social domains (home, work, and social events), following the framework proposed by Gullifer and Titone (2020). Together, these measures provide a broad and cohesive account of bilingual experience, enabling us to examine how these linguistic factors could predict task-switching performance in bilinguals. We hypothesize that greater diversity and balance in bilingual language use, reflected in higher language entropy and more frequent or contextually varied language switching, will be associated with enhanced cognitive flexibility, reflected as smaller mix and switch costs on task-switching performance. In addition, because MAD provides a continuous index of response dynamics and is therefore potentially more sensitive than RT to subtle variations in cognitive control, we hypothesize that effects of bilingual experience will be easier to be detected in MAD than in RT for both switch and mix costs, reflecting finer-grained differences in the efficiency of conflict monitoring and resolution processes.

## 2. Methods

### 2.1. Participants

Fifty-one Chinese–English bilinguals were recruited online and reimbursed for their time. All participants confirmed that they had normal or corrected-to-normal vision, and were comfortable using a computer mouse with their right hand. Ethical approval for this study was granted by Institutional Review Board of Faculty of Psychology, Southwest University (No. H23042).

### 2.2. Materials and Procedures

#### 2.2.1. Colour-Shape Task Switching Paradigm

**Figure 1:**
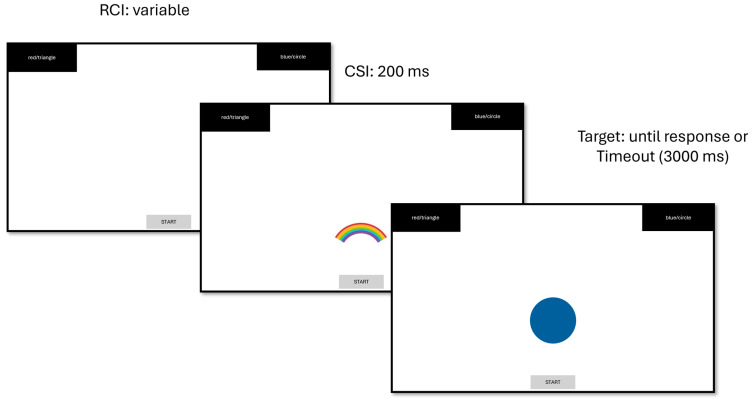
The procedure of colour-shape task switching paradigm in the present study.

The paradigm used in this study is a typical colour-shape task switching paradigm (Prior & MacWhinney, 2010; see Figure 1). In each trial, participants are instructed to judge a presented coloured shape based on either colour (red/blue) or shape (triangle/circle), depending on a cue. In a pure block, participants judged only one dimension of the stimulus (either colour or shape). In a mixed block, both sets of cues were presented, and participants had to judge the relevant dimension based on the cue. There was a total of 198 trials, including 42 practice trials, two pure blocks (one colour and one shape) of 60 trials each, and one mix block of 96 trials. The trials were pseudo-randomized between colour and shape decisions, resulting in equal probabilities for task switch (switch vs. repeat). Feedback on inaccurate responses was provided to participants throughout the experimental session.

The experiment was conducted on Gorilla (http://gorilla.sc; Anwyl-Irvine et al., 2020). As a mouse tracking study, participants initiated a trial by clicking on a Start button at the bottom of the screen. A task cue then appeared in the lower half of the screen, and two response buttons appeared in the top-left and top-right corners. For the colour task, the cue was either a stylised rainbow or a colour wheel; for the shape task, the cue was either a horizontal display of a circle, square, and triangle, or a circle embedded within a triangle, itself embedded within a square.

Cue-stimulus interval was 200 ms, and two cue sets alternated for each task, ensuring that cues for adjacent trials never repeated, thereby avoiding potential cue repetition effects. After 200 ms, the cue was replaced with the target stimulus (e.g., a red triangle). Participants then moved their mouse from the start area toward the appropriate response field (i.e., top-left or top-right corner) and clicked on it to complete a trial. If no response was made within 3000 ms, the target stimulus disappeared, and the trial skipped to next one; this cutoff was chosen to provide participants sufficient time to respond while preventing excessively long trials that could reflect inattention or fatigue. As response repetition effect often interacts with task switching, we included response repetition (0 = different, 1 = same) as one of the predictors.

#### 2.2.2. Questionnaires

We applied a modified version of the LSBQ (Anderson et al., 2018), LHQ 2.0 (P. Li et al., 2014) and LEAP-Q (Marian et al., 2007) to capture participants’ demographics and language variables. Participants completed the questionnaire after the switching task. The following demographic variables were selected: *gender* (0 = male, 1 = female), *age in years*, *education* on a 7-point scale (1 = no formal education, 2 = did not graduate from high school/no G.C.S.E.s, 3 = graduated from high school/G.C.S.E.s, 4 = graduated from college/6th form, 5 = undergraduate degree, 6 = graduate or professional degree beyond a bachelor’s, e.g., MPhil, MSc, 7 = doctoral degree, e.g., PhD, MD), *socio-economic status (SES)*, calculated as the average of father’s education, mother’s education, and family income, each rated on a 5-point scale.

For language-related variables, the predictors were derived from three questionnaires as described earlier, with details provided below.

*LSBQ. Composite Factor Scores (CFS)*, reflecting overall level of bilingualism, was calculated from multiple LSBQ items, including L2 home use and proficiency, L2 social use and L1 proficiency (see more details in Anderson et al., 2018); and *language use across situations/people/life stages/activities*, which was assessed on a 5-point scale (0 = exclusively Language 1, 1 = mostly Language 1, 2 = half Language 1/half other language, 3 = mostly the other language, 4 = exclusively the other language). For analysis, these scores were recoded, with the original scores 3 and 4 flipped to 0 and 1, respectively, so that lower values reflected greater use of one language (L1 or L2), while higher values reflected a more balanced use of two languages; *Switching with family/with friends/on social media* on a 5-point scale (0 = never, 1 = rarely, 2 = sometimes, 3 = frequently, 4 = always).

*LHQ 2.0 and LEAP-Q. Age of bilingualism in years*, and *language entropy at home/work/social events* (representing diversity in language use across two languages), which was calculated from language exposure in different usage contexts (Gullifer & Titone, 2020) and using *languageEntropy* package (Gullifer et al., 2018) in R 4.3.0 (R Core Team, 2023).

### 2.3. Data Preprocessing

Data were processed in R 4.3.0 (R Core Team, 2023) using the packages *mousetrap* (Kieslich & Henninger, 2017), and *lmerTest* (Kuznetsova et al., 2017). The raw mouse trajectories were time-normalized using the function mt_time_normalize() from the *mousetrap* package, which resampled each trajectory into 101 time steps using linear interpolation to ensure comparability across trials of different durations. For each response, response accuracy, reaction time (RT, i.e., the time interval between onset of the stimulus display, and clicking on the response field), and MAD (i.e., largest perpendicular deviation of the actual mouse trajectory from the ideal straight line, measured in pixels; Wulff et al., 2021), were computed. Six participants were excluded due to data loss or incomplete data, resulting in a final sample of 45 participants for data analysis. In addition, trials with recording errors or extremely short trajectories were removed. Due to high accuracy (92.1%), incorrect responses were removed from analysis, and errors were not further analyzed.

## 3. Results

Multiple regression analyses were conducted to examine how a range of demographic, cognitive, and language-related factors predicted performance on task switching measures. Specifically, we analyzed both switch costs and mix costs in RT and MAD. Switch cost for each participant was computed as the difference in mean performance between switch and repeat trials within mixed blocks (*switch cost* = *switch* − *repeat*), and mix cost for each participant was calculated as the difference in the mean performance between mixed blocks and pure blocks (*mix cost* = *mixed* − *pure).* The full model comprised all factors, including gender, age, education, SES, response repetition, CFS, age of bilingualism, language switching with family/with friends/on social media, language entropy at home/work/social events, and language use across situations/people/life stages/activities. Model comparison was conducted between this full model and a null model that included only the intercept. Any data rows containing missing values on variables entered in the model were automatically excluded from the analysis. See Table 1 for the summary of the data. In addition, stepwise selection was employed to identify the best-performing model. The estimates from the full model are presented in the following tables (from Table 2, Table 3, Table 4 and Table 5), whereas the estimates from the reduced model selected through stepwise selection are reported in the main text. 

### 3.1. Mix Costs in RT

The multiple regression on mix cost RT explained 24% of the variance in the full model (adj. R^2^ = 0.24; see Table 2). Model comparison indicated that the full model accounted for significantly more variance than the null model, *F*(17, 48) = 2.21, *p* = 0.017. In the reduced model selected through stepwise regression, language use across life stages (*β* = −222.18, *p* < 0.001), language use across activities (*β* = −275.31, *p* < 0.001), CFS (*β* = 43.71, *p* < 0.001), language entropy at work (*β* = 158.23, *p* = 0.013), language switching with friends (*β* = −133.27, *p* = 0.001), and language switching on social media (*β* = 130.07, *p* = 0.013) were significant predictors. Language switching with family (*β* = −89.34, *p* = 0.064) was retained but not significant. This stepwise model explained 33% of the variance and showed improved fit relative to both the null model and the full model, with the lowest AIC (886.50) and BIC (906.20).

**Table 2:** Multiple Regression for the Full Model Predicting Mix Cost RT.

Predictor	*β*	*SE*	*t* Values	*p*
Intercept	423.94 [−773.58, 1621.47]	595.59	0.71	0.480
*Basic demographics*				
Gender (0 = male, 1 = female)	2.11 [−142.88, 147.09]	72.11	0.03	0.977
Age	0.52 [−46.48, 47.51]	23.37	0.02	0.982
Education	−11.37 [−85.09, 62.34]	36.66	−0.31	0.758
SES	24.38 [−38.68, 87.44]	31.36	0.78	0.441
Age of bilingualism	5.90 [−11.88, 23.69]	8.84	0.67	0.508
*Overall level of bilingualism*				
CFS	47.46 [14.24, 80.67]	16.52	2.87	0.006 **
*Language switching*				
Switching family	−76.48 [−206.64, 53.68]	64.74	−1.18	0.243
Switching friends	−122.99 [−228.41, −17.57]	52.43	−2.35	0.023 *
Switching social media	129.81 [−34.31, 293.94]	81.63	1.59	0.118
*Language entropy*				
Language entropy at home	−25.72 [−174.50, 123.06]	74.00	−0.35	0.730
Language entropy at work	169.05 [−181.63, 519.72]	174.41	0.97	0.337
Language entropy at social events	−7.77 [−358.54, 343.00]	174.46	−0.05	0.965
*Language usage*				
Language use situations	−98.19 [−440.62, 244.24]	170.31	−0.58	0.567
Language use people	−8.49 [−207.94, 190.96]	99.20	−0.09	0.932
Language use life stages	−192.98 [−371.66, −14.29]	88.87	−2.17	0.035 *
Language use activities	−236.00 [−436.80, −35.20]	99.87	−2.36	0.022 *
*Response repetition* (0 = different, 1 = same)	49.66 [−48.16, 147.49]	48.65	1.02	0.312
**Model-Level Statistics**				
**Model**	**Predictors**	**Adj. R^2^**	**AIC**	**BIC**
Null	Intercept only	0.00	906.19	910.57
Full	All predictors	0.24	902.09	943.69

Note. * *p* < 0.05, ** *p* < 0.01.

To further explore the potential collinearity among predictors, we used the car package (Fox & Weisberg, 2018) in R to calculate variance inflation factors (VIF) for the full model. The results indicated that most predictors fell well below the conventional threshold of concern (VIFs < 5), suggesting limited multicollinearity for the majority of variables. However, three predictors, language entropy at work (VIF = 8.94), language entropy at social events (VIF = 9.69), and language use across situations (VIF = 9.35), showed elevated values approaching 10, indicating substantial redundancy with other predictors in the model. These findings suggest that while multicollinearity was not a general concern, the high overlap among these three predictors should be taken into consideration when interpreting their coefficients.

The estimates from the full multiple regression model indicated that CFS, switching with friends, and language use across life stages and activities emerged as significant predictors of mix cost RT (see Table 2 for details). Importantly, these four predictors were also retained in the best-fitting model identified through stepwise selection, suggesting that they represent robust factors that contribute to variance in mix cost RT across different model specifications. In addition to these predictors, language switching on social media, language switching with family, and language entropy at work were also included in the reduced stepwise model. Notably, among these three factors, language entropy at work displayed a high variance inflation factor (VIF > 5), indicating multicollinearity with other predictors. This redundancy likely explains why it was not significant in the full model but nonetheless appeared in the stepwise solution, where correlated variables were selectively dropped.

### 3.2. Mix Costs in MAD

The full multiple regression model on mix cost MAD accounted for a substantial proportion of variance, adj. R^2^ = 0.40 (see Table 3), and provided a significantly better fit than the null model, *F*(17, 48) = 3.59, *p* < 0.001. In the reduced model selected through stepwise selection, age (*β* = −16.59, *p* = 0.014), education (*β* = 36.34, *p* = 0.001), age of bilingualism (*β* = −7.11, *p* = 0.005), CFS (*β* = 31.30, *p* < 0.001), language switching on social media (*β* = −68.20, *p* < 0.001), language use across people (*β* = −126.20, *p* < 0.001), and language use across activities (*β* = −63.13, *p* = 0.034) emerged as significant predictors, while language use across situations (*β* = −61.84, *p* = 0.115) was retained but not significant. This stepwise model explained 46% of the variance and showed improved fit relative to both the null model and the full model, with the lowest AIC (757.58) and BIC (779.47).

**Table 3:** Multiple Regression for the Full Model Predicting Mix Cost MAD.

Predictor	*β*	*SE*	*t* Values	*p*
Intercept	498.96 [57.98, 939.94]	219.32	2.28	0.027 *
*Basic demographics*				
Gender (0 = male, 1 = female)	8.95 [−44.44, 62.33]	26.55	0.34	0.738
Age	−17.42 [−34.72, −0.11]	8.61	−2.02	0.049 *
Education	43.47 [16.32, 70.61]	13.50	3.22	0.002 **
SES	6.55 [−16.67, 29.77]	11.55	0.57	0.573
Age of bilingualism	−6.33 [−12.88, 0.22]	3.26	−1.94	0.058
*Overall level of bilingualism*				
CFS	28.80 [16.56, 41.03]	6.08	4.73	<0.001 ***
*Language switching*				
Switching family	−15.69 [−63.62, 32.24]	23.84	−0.66	0.514
Switching friends	10.58 [−28.24, 49.40]	19.31	0.55	0.586
Switching social media	−59.46 [−119.90, 0.97]	30.06	−1.98	0.054
*Language entropy*				
Language entropy at home	−4.13 [−58.92, 50.65]	27.25	−0.15	0.880
Language entropy at work	20.66 [−108.47, 149.80]	64.23	0.32	0.749
Language entropy at social events	−45.34 [−174.51, 83.83]	64.24	−0.71	0.484
*Language usage*				
Language use situations	−29.76 [−155.85, 96.34]	62.72	−0.47	0.637
Language use people	−135.47 [−208.92, −62.02]	36.53	−3.71	<0.001 ***
Language use life stages	−13.29 [−79.08, 52.51]	32.73	−0.41	0.687
Language use activities	−57.77 [−131.72, 16.17]	36.78	−1.57	0.123
*Response repetition* (0 = different, 1 = same)	22.25 [−13.77, 58.27]	17.92	1.24	0.220
**Model-Level Statistics**				
**Model**	**Predictors**	**Adj. R^2^**	**AIC**	**BIC**
Null	Intercept only	0.00	790.35	794.73
Full	All predictors	0.40	770.22	811.82

Note. * *p* < 0.05, ** *p* < 0.01, *** *p* < 0.001.

As indicated in Table 3, the full model estimation for mix cost MAD revealed significant effects of participants’ age, education, CFS, and language use across people, with age of bilingualism and language switching on social media emerging as marginally significant predictors. All of these variables were retained in the best-fitting model identified through stepwise selection, which also included several additional predictors. The earlier VIF analysis showed relatively high multicollinearity for language entropy at work and at social events, as well as for language use across situations. Taken together, these results indicate that age, education, CFS, and language use across people are robust contributors to mix cost MAD, while the extra factors selected only in the stepwise model may reflect overlapping variance caused by multicollinearity rather than unique, independent effects.

### 3.3. Switch Costs in RT

A multiple regression was conducted to examine predictors of switch cost RT. As shown in Table 4, the full model did not account for significantly more variance in switch cost RT than the null model (*F*(17, 48) = 1.36, *p* = 0.20, adj. R^2^ = 0.09). A stepwise regression retained only response repetition as a significant predictor (*β* = 86.34, *p* = 0.003), indicating that switch costs were higher on repeated-response trials compared with non-repeated trials. This reduced model accounted for 12% of the variance in switch cost RT and showed improved model fit compared with both the null model and full model, with the lowest AIC (813.81) and BIC (820.38).

**Table 4:** Multiple Regression for the Full Model Predicting Switch Cost RT.

Predictor	*β*	*SE*	*t* Values	*p*
Intercept	748.96 [59.28, 1438.63]	343.01	2.18	0.034 *
*Basic demographics*				
Gender (0 = male, 1 = female)	−22.62 [−106.12, 60.88]	41.53	−0.55	0.588
Age	−19.78 [−46.84, 7.29]	13.46	−1.47	0.148
Education	−8.63 [−51.08, 33.82]	21.12	−0.41	0.685
SES	25.52 [−10.80, 61.83]	18.06	1.41	0.164
Age of bilingualism	−5.66 [−15.90, 4.58]	5.09	−1.11	0.272
*Overall level of bilingualism*				
CFS	7.92 [−11.21, 27.05]	9.51	0.83	0.409
*Language switching*				
Switching family	52.63 [−22.33, 127.59]	37.28	1.41	0.165
Switching friends	−12.97 [−73.68, 47.74]	30.20	−0.43	0.669
Switching social media	−121.52 [−216.04, −27.00]	47.01	−2.59	0.013 *
*Language entropy*				
Language entropy at home	93.09 [7.40, 178.77]	42.62	2.18	0.034 *
Language entropy at work	37.18 [−164.78, 239.14]	100.45	0.37	0.713
Language entropy at social events	−81.78 [−283.80, 120.23]	100.47	−0.81	0.420
*Language usage*				
Language use situations	−80.05 [−277.26, 117.16]	98.08	−0.82	0.418
Language use people	3.91 [−110.95, 118.78]	57.13	0.07	0.946
Language use life stages	−45.30 [−148.20, 57.61]	51.18	−0.89	0.381
Language use activities	49.95 [−65.69, 165.60]	57.52	0.87	0.389
*Response repetition* (0 = different, 1 = same)	86.34 [30.00, 142.68]	28.02	3.08	0.003 **
**Model-Level Statistics**				
**Model**	**Predictors**	**Adj. R^2^**	**AIC**	**BIC**
Null	Intercept only	0.00	821.26	825.64
Full	All predictors	0.09	829.25	870.85

Note. * *p* < 0.05, ** *p* < 0.01.

As shown in Table 4, the full model estimation revealed significant effects of language switching on social media, language entropy at home, and response repetition. However, the stepwise model selection retained only response repetition as a significant predictor, indicating that the additional variance explained by language switching on social media and language entropy at home did not improve overall model fit enough to justify the added complexity. Taken together, these results point to response repetition as the most robust and reliable predictor of switch cost RT, whereas the effects of language switching on social media and language entropy at home appear weaker or partly redundant and therefore require cautious interpretation.

### 3.4. Switch Costs in MAD

A multiple regression was conducted to examine predictors of switch cost MAD. The full model accounted for significantly more variance than the null model (*F*(17, 48) = 2.08, *p* = 0.024), explaining approximately 22% of the variance (adj. R^2^ = 0.22; see Table 5). As shown in Table 5, the full model estimation for switch cost MAD revealed significant effects of gender, language switching with family, language switching on social media, language entropy at home, and language use across both situations and activities. For the stepwise model, the best-fitting model (adj. R^2^ = 0.21, AIC = 965.32, BIC = 978.46) retained gender (*β* = −303.39, *p* = 0.002), language use across situations (*β* = −430.40, *p* = 0.005), and age (*β* = 69.37, *p* = 0.014) as significant predictors, with language use across activities (*β* = 215.26, *p* = 0.098) showing only marginally significant. The earlier VIF analysis indicated relatively high multicollinearity for language entropy at work and social events, as well as for language use across situations. Taken together, these findings identify gender and language use across situations as the most robust predictors of switch cost MAD. While the additional effects observed in the full model, particularly those involving language entropy, may reflect overlapping variance and should therefore be interpreted with caution.

**Table 5:** Multiple Regression for the Full Model Predicting Switch Cost MAD.

Predictor	*β*	*SE*	*t* Values	*p*
Intercept	88.82 [−1986.82, 2164.45]	1032.33	0.09	0.932
*Basic demographics*				
Gender (0 = male, 1 = female)	−332.87 [−584.17, −81.58]	124.98	−2.66	0.011 *
Age	28.48 [−52.97, 109.93]	40.51	0.70	0.485
Education	−60.24 [−188.01, 67.53]	63.55	−0.95	0.348
SES	77.18 [−32.12, 186.48]	54.36	1.42	0.162
Age of bilingualism	−25.44 [−56.27, 5.38]	15.33	−1.66	0.103
*Overall level of bilingualism*				
CFS	9.41 [−48.16, 66.98]	28.63	0.33	0.744
*Language switching*				
Switching family	226.31 [0.70, 451.91]	112.21	2.02	0.049 *
Switching friends	−24.65 [−207.37, 158.07]	90.88	−0.27	0.787
Switching social media	−412.79 [−697.26, −128.31]	141.49	−2.92	0.005 **
*Language entropy*				
Language entropy at home	268.35 [10.47, 526.22]	128.26	2.09	0.042 *
Language entropy at work	−448.65 [−1056.47, 159.17]	302.30	−1.48	0.144
Language entropy at social events	296.83 [−311.14, 904.81]	302.38	0.98	0.331
*Language usage*				
Language use situations	−863.74 [−1457.26, −270.21]	295.19	−2.93	0.005 **
Language use people	103.29 [−242.41, 448.98]	171.94	0.60	0.551
Language use life stages	61.80 [−247.91, 371.51]	154.04	0.40	0.690
Language use activities	454.05 [106.00, 802.09]	173.10	2.62	0.012 *
*Response repetition* (0 = different, 1 = same)	84.22 [−85.34, 253.77]	84.33	1.00	0.323
**Model-Level Statistics**				
**Model**	**Predictors**	**Adj. R^2^**	**AIC**	**BIC**
Null	Intercept only	0.00	977.10	981.48
Full	All predictors	0.22	974.69	1016.29

Note. * *p* < 0.05, ** *p* < 0.01.

## 4. Discussion

The present study aims to investigate the factors predicting cognitive flexibility in Chinese–English bilinguals, with particular attention to the comprehensive inclusion of demographic, language proficiency, and language switching variables. Cognitive flexibility was assessed using both RTs and MAD derived from a mouse-tracking nonverbal task-switching paradigm, which included measures of both mix costs and switch costs. Our analyses indicate that the selected predictors account for a larger proportion of the variance in mix costs than in switch costs, with this effect being particularly pronounced for MAD compared to RTs. These findings suggest that bilingual experience may be more strongly associated with the sustained control processes, rather than with the transient control processes.

For mix costs, several consistent patterns emerged across both RT and MAD, including CFS (represent overall bilingualism level by integrating self-reported proficiency and everyday language use into a single global index; Anderson et al., 2018), and measures of language use across contexts. Higher CFS was positively associated with mix costs, suggesting that individuals with higher overall bilingualism scores showed greater costs when maintaining two task sets. In contrast, measures of language use (across life stages and activities for RTs; across people for MAD) were negatively associated with mix costs. That is, while overall bilingual proficiency (as indexed by CFS) may be associated with increased demands during sustained control, a more balanced pattern of language use appears to mitigate these costs.

Previous studies exploring bilingualism level typically report reduced or unchanged mix costs with greater bilingual experience. For example, Yow and Li (2015) found that participants who acquired their second language early and maintained more balanced use between their two languages demonstrated smaller mix costs in task-switching paradigms. Extending this line of inquiry, Wang et al. (2022) examined how second-language proficiency interacts with domain-general cognitive control. They showed that higher L2 proficiency altered the relationship between mix costs and inhibitory control, with stronger associations emerging between mix costs and Simon task performance among highly proficient bilinguals, although L2 proficiency itself did not directly predict the magnitude of mix costs. In addition, Tse and Altarriba (2014, 2015) used vocabulary and fluency measures in Cantonese-English bilinguals, and observed that neither first- nor second-language proficiency reliably predicted mix costs, although higher second-language proficiency sometimes yielded marginal benefits in local switching or conflict resolution.

One possible explanation lies in the composition of the CFS itself. Because this score integrates L2 home use and proficiency, L2 social use, and L1 proficiency into a single index (Anderson et al., 2018; DeLuca et al., 2019; Luk & Bialystok, 2013), high CFS values may partly reflect intensive language switching and rich interactional contexts rather than balanced competence alone. In other words, its multidimensional nature makes it difficult to disentangle which component drives its association with mix costs. To disentangle these effects, a follow-up hierarchical regression analyses were conducted on mix cost in both RT and MAD, focusing on the three factors comprising the CFS: L2 home use and proficiency, L2 social use, and L1 proficiency. All predictors were z-standardized prior to analysis, and model comparisons were used to evaluate the incremental variance explained at each step. For mix cost in RT, adding L2 home use and proficiency significantly improved model fit, *F*(1, 64) = 7.80, *p* < 0.01, and was a positive predictor (*β* = 72.20, *p* < 0.05). In contrast, L2 social use and L1 proficiency did not improve the model or reach significance (*p* > 0.10). A similar pattern emerged in MAD: only L2 home use and proficiency significantly enhanced model fit, *F*(1, 64) = 7.91, *p* = 0.007, and predicted higher MAD (*β* = 37.49, *p* < 0.01), whereas the other factors did not improve the model fit and remained non-significant (*p* > 0.11). That is, higher L2 home use and proficiency predicted larger mix costs in both RT and MAD. This pattern of results suggests that within the CFS, L2 home use and proficiency were the primary contributors to mix costs in both RT and MAD. Although counterintuitive, these findings might highlight that language use at home could be a specific situation that positively predicts mix costs.

Furthermore, it is also important to note that although the CFS showed a positive association with mix costs, several specific measures of language use displayed the opposite and expected pattern. In the present study, language use across life stages and activities (for RTs) and language use across people (for MAD) were negatively associated with mix costs, indicating that a balanced and diversified pattern of everyday language use may help offset the proactive demands. In line with this evidence, Zhou and Krott (2015) found that balanced bilingual proficiency, indicated by comparable performance on vocabulary and fluency tasks in both languages and reflecting broad use of each language across contexts, was associated with smaller mix costs. This pattern is consistent with previous evidence showing that frequent and contextually varied language use supports cognitive flexibility and efficient control (DeLuca et al., 2019; Gullifer & Titone, 2020).

These findings highlight the broader challenge of defining and operationalizing bilingual experience, a point repeatedly emphasized in recent methodological critiques (e.g., Anderson et al., 2018; DeLuca et al., 2019; Luk & Bialystok, 2013). Because bilingualism spans proficiency, use, switching, age of acquisition, and sociocultural context, a single composite score can mask distinct effects. Some facets, such as high competence combined with intense switching, may elevate global costs, whereas balanced, contextually rich language use may buffer against them. This pattern reinforces calls to move beyond global factor scores and adopt multi-dimensional, experience-based approaches (e.g., DeLuca et al., 2020; Surrain & Luk, 2019) so that future work can clarify how specific aspects of bilingual experience jointly shape proactive and reactive control.

The analyses of switch costs revealed a pattern distinct from that observed for mix costs. For switch costs in RTs, the full model explained only a small and non-significant portion of variance, and the stepwise regression retained only response repetition as a significant predictor. No language-related variables were identified as predictors for switch costs in RTs, which is consistent with the null findings in previous research (Lehtonen et al., 2018; Paap et al., 2015; Timmermeister et al., 2020; Zhu et al., 2025). In contrast, for switch costs in MAD, the full model accounted for more variance, and language use across situations was identified as a significant predictor in both full and stepwise models. More specifically, a more balanced pattern of language use across situational contexts was associated with reduced switch costs. This fits with the Adaptive Control Hypothesis: bilinguals frequently using both languages in the same context (dual-language context) train their control mechanisms more. For instance, Hartanto and Yang (2016) showed that bilinguals in a dual-language context had significantly smaller switch costs than those in single-language contexts. Thus, the present results suggest that a generally balanced pattern of language use across different contexts (e.g., home, school, work) is related to enhanced task-switching performance. These findings may also indicate that measures of movement curvature derived from mouse tracking are more sensitive and informative than response latencies (Freeman et al., 2010; Hehman et al., 2015).

Other noteworthy predictors also deserve detailed consideration. Age of bilingualism emerged as marginally significant in the full model estimates of mix costs in MAD yet was retained in the stepwise-selected model, suggesting some predictive value after accounting for model complexity. Interestingly, the estimate was negative, indicating that individuals who became bilingual later in life exhibited smaller mix costs in MAD. This direction is counterintuitive because earlier bilingual acquisition is typically associated with greater and more prolonged experience in managing two languages, which many theories link to better executive control (e.g., Khodos et al., 2021; Khodos & Moskovsky, 2020; Luk et al., 2011; Gullifer et al., 2018). It is possible that later bilinguals may use their second language more selectively, in well-defined contexts that foster efficient, context-specific control rather than continuous monitoring. In addition, Khodos and Moskovsky (2020) distinguished between the age at which second-language acquisition began and the onset age of active bilingualism, whereas our study did not separate these questions, so participants may have interpreted “age of bilingualism” differently. Overall, it is important to note, even though age of bilingualism was retained in the stepwise-selected model, it was not significant in the current study of the full model. Therefore, we interpret this effect cautiously and do not treat it as robust evidence, and future research should clarify how these distinct age measures influence cognitive control.

Two language-switching measures also showed noteworthy associations. Switching with friends was negatively associated with mix costs in RT, implying that frequent informal switching in social interactions may strengthen the ability to maintain multiple task sets efficiently, consistent with accounts that emphasize the cognitive benefits of rich, socially embedded language use (Gullifer & Titone, 2020). Switching on social media was marginally significant in the full model for mix costs in MAD and was retained in the stepwise model, again with a negative coefficient, pointing to a similar facilitative trend. Social-media contexts often require rapid, flexible alternation between languages and communicative norms, which could promote the proactive monitoring and interference control needed to reduce global costs (Androutsopoulos, 2015; Green & Abutalebi, 2013; Gullifer & Titone, 2020; Tiv et al., 2020).

In addition, language entropy at home showed a positive association with switch costs in both RT and MAD in the estimation of full models, although it was not retained in the stepwise-selected models. This indicates that greater diversity of home-language use (higher entropy) was linked to larger, rather than smaller, costs when alternating between task sets. The direction is somewhat unexpected, because previous studies typically report neutral or negative (i.e., beneficial) relations between language entropy and executive control (Beatty-Martínez & Dussias, 2017; Gullifer et al., 2018; Gullifer & Titone, 2020; Hartanto & Yang, 2016; Tiv et al., 2020). One possible explanation is that high home-language entropy may create frequent and unpredictable demands for language control, which in turn could increase the need for reactive control during task switching and thereby inflate switch costs. Although this interpretation should be viewed with caution, the finding that higher home-language entropy was associated with larger switch costs may suggest that constant alternation between languages at home does not necessarily train reactive control in a way that benefits non-verbal switching tasks. Instead, it might impose situational demands that do not transfer straightforwardly to laboratory measures of task switching. Interestingly, both language entropy at home and L2 home use and proficiency (one component of CFS) positively predicted task switching, which might suggest that language experiences in the home environment may influence cognitive flexibility in distinct ways. Further studies will be needed to clarify whether the present effect reflects a sample-specific pattern, a task-specific sensitivity, or a boundary condition on the proposed benefits of entropy.

Finally, several predictors in our models showed substantial redundancy with other variables (i.e., language entropy at work, language entropy at social events, and language use across situations), reflecting the conceptual overlap inherent in bilingualism assessments. Although multicollinearity did not pose a statistical concern, this redundancy highlights that many language measures capture related aspects of bilingual experience. Consequently, the unique variance explained by any single predictor may be small, and the observed effects likely reflect shared aspects of bilingual experience rather than isolated features. Future work could consider composite indices or latent factors to more parsimoniously represent overlapping language dimensions.

## 5. Conclusions

The present study provided a comprehensive assessment of cognitive flexibility in Chinese–English bilinguals by jointly examining language proficiency- and language switching-related variables together with key demographic factors, and by analyzing both reaction times (RTs) and movement curvature (MAD) derived from a mouse-tracking task-switching paradigm. Overall, our results indicate that bilingual experience is more strongly associated with sustained control processes (mix costs) than with transient control processes (switch costs), particularly when measured via movement trajectories. Bilingual experience influences cognitive flexibility in multiple ways. While higher overall proficiency may be correlated with increased demands on sustained control, balanced and diverse language use appears to mitigate these costs and support more efficient task switching. Moreover, bilingual experiences in the home context emerged as a potential factor positively predicting cognitive flexibility.

## Figures and Tables

**Table 1:** Participant language information.

	Sample (N = 45, Male = 16, Female = 29)			
	M	*SD*	Min	Max
*Basic demographics*				
Age (years)	21.60	1.56	19	26
Education (1–7)	4.71	0.91	2	6
SES (1–5)	2.65	1.01	1.33	4.67
Age of bilingualism (years)	13.80	4.18	4	22
*Language switching (0* *–* *4)*				
Switching with family	0.81	0.93	0	4
Switching with friends	1.84	1.02	0	4
Switching on social media	1.74	0.76	0	4
*Overall level of bilingualism*				
CFS	0.71	3.15	−4.42	8.93
*Language entropy*				
Language entropy at home	0.35	0.50	−0.40	1
Language entropy at work	0.65	0.42	−0.40	1
Language entropy at social events	0.60	0.42	−0.55	1
*Language usage (0* *–* *2)*				
Language use across situations	0.51	0.44	0	1.38
Language use across people	0.48	0.41	0	1.25
Language use across life stages	0.70	0.53	0	2
Language use across activities	0.75	0.51	0	2

## Data Availability

Data is available on request from the first author.

## Abbreviations

The following abbreviations are used in this manuscript:

CFS	Composite factor score
LEAP-Q	Language Experience and Proficiency Questionnaire
LHQ	Language History Questionnaire
LSBQ	Language and Social Background Questionnaire
MAD	Maximum absolute deviation
RT	Reaction time
SES	Socio-economic status
VIF	Variance inflation factors

## References

[B1-behavsci-15-01481] Anderson J. A. E., Mak L., Keyvani Chahi A., Bialystok E. (2018). The language and social background questionnaire: Assessing degree of bilingualism in a diverse population. Behavior Research Methods.

[B2-behavsci-15-01481] Androutsopoulos J. (2015). Networked multilingualism: Some language practices on Facebook and their implications. International Journal of Bilingualism.

[B3-behavsci-15-01481] Anwyl-Irvine A. L., Dalmaijer E. S., Hodges N., Evershed J. K. (2020). Online experimentation using gorilla. Behavior Research Methods.

[B4-behavsci-15-01481] Beatty-Martínez A. L., Dussias P. E. (2017). Bilingual experience shapes language processing: Evidence from codeswitching. Journal of Memory and Language.

[B5-behavsci-15-01481] Bialystok E. (2017). The bilingual adaptation: How minds accommodate experience. Psychological Bulletin.

[B6-behavsci-15-01481] Braver T. S., Reynolds J. R., Donaldson D. I. (2003). Neural mechanisms of transient and sustained cognitive control during task switching. Neuron.

[B7-behavsci-15-01481] de Bruin A. (2019). Not all bilinguals are the same: A call for more detailed assessments and descriptions of bilingual experiences. Behavioral Sciences.

[B8-behavsci-15-01481] DeLuca V., Rothman J., Bialystok E., Pliatsikas C. (2019). Redefining bilingualism as a spectrum of experiences that differentially affects brain structure and function. Proceedings of the National Academy of Sciences.

[B9-behavsci-15-01481] DeLuca V., Segaert K., Mazaheri A., Krott A. (2020). Understanding bilingual brain function and structure changes? U bet! A unified bilingual experience trajectory model. Journal of Neurolinguistics.

[B10-behavsci-15-01481] Fox J., Weisberg S. (2018). An R companion to applied regression.

[B11-behavsci-15-01481] Freeman J. B., Ambady N. (2010). MouseTracker: Software for studying real-time mental processing using a computer mouse-tracking method. Behavior Research Methods.

[B12-behavsci-15-01481] Freeman J. B., Pauker K., Apfelbaum E. P., Ambady N. (2010). Continuous dynamics in the real-time perception of race. Journal of Experimental Social Psychology.

[B13-behavsci-15-01481] Green D. W., Abutalebi J. (2013). Language control in bilinguals: The adaptive control hypothesis. Journal of Cognitive Psychology.

[B14-behavsci-15-01481] Grundy J. G., Anderson J. A. E., Bialystok E. (2017). Neural correlates of cognitive processing in monolinguals and bilinguals. Annals of the New York Academy of Sciences.

[B15-behavsci-15-01481] Gullifer J. W., Chai X. J., Whitford V., Pivneva I., Baum S., Klein D., Titone D. (2018). Bilingual experience and resting-state brain connectivity: Impacts of L2 age of acquisition and social diversity of language use on control networks. Neuropsychologia.

[B16-behavsci-15-01481] Gullifer J. W., Titone D. (2020). Characterizing the social diversity of bilingualism using language entropy. Bilingualism: Language and Cognition.

[B17-behavsci-15-01481] Hartanto A., Yang H. (2016). Disparate bilingual experiences modulate task-switching advantages: A diffusion-model analysis of the effects of interactional context on switch costs. Cognition.

[B18-behavsci-15-01481] Hartanto A., Yang H. (2020). The role of bilingual interactional contexts in predicting interindividual variability in executive functions: A latent variable analysis. Journal of Experimental Psychology: General.

[B19-behavsci-15-01481] Hehman E., Stolier R. M., Freeman J. B. (2015). Advanced mouse-tracking analytic techniques for enhancing psychological science. Group Processes & Intergroup Relations.

[B20-behavsci-15-01481] Khodos I., Moskovsky C. (2020). Dimensions of bilingualism promoting cognitive control: Impacts of language context and onset age of active bilingualism on mixing and switching costs. Linguistic Approaches to Bilingualism.

[B21-behavsci-15-01481] Khodos I., Moskovsky C., Paolini S. (2021). Bilinguals’ and monolinguals’ performance on a non-verbal cognitive control task: How bilingual language experience contributes to cognitive performance by reducing mixing and switching costs. International Journal of Bilingualism.

[B22-behavsci-15-01481] Kiesel A., Steinhauser M., Wendt M., Falkenstein M., Jost K., Philipp A. M., Koch I. (2010). Control and interference in task switching—A review. Psychological Bulletin.

[B23-behavsci-15-01481] Kieslich P. J., Henninger F. (2017). Mousetrap: An integrated, open-source mouse-tracking package. Behavior Research Methods.

[B24-behavsci-15-01481] Kroll J. F., Bialystok E. (2013). Understanding the consequences of bilingualism for language processing and cognition. Journal of Cognitive Psychology.

[B25-behavsci-15-01481] Kuznetsova A., Brockhoff P. B., Christensen R. H. B. (2017). lmerTest package: Tests in linear mixed effects models. Journal of Statistical Software.

[B26-behavsci-15-01481] Laine M., Lehtonen M. (2018). Cognitive consequences of bilingualism: Where to go from here?. Language, Cognition and Neuroscience.

[B27-behavsci-15-01481] Lehtonen M., Soveri A., Laine A., Järvenpää J., de Bruin A., Antfolk J. (2018). Is bilingualism associated with enhanced executive functioning in adults? A meta-analytic review. Psychological Bulletin.

[B28-behavsci-15-01481] Li P., Zhang F., Tsai E., Puls B. (2014). Language history questionnaire (LHQ 2.0): A new dynamic web-based research tool. Bilingualism: Language and Cognition.

[B29-behavsci-15-01481] Li X., Ng K. K., Wong J. J. Y., Lee J. W., Zhou J. H., Yow W. Q. (2021). Bilingual language entropy influences executive functions through functional connectivity and signal variability. Brain and Language.

[B30-behavsci-15-01481] Los S. A. (1996). On the origin of mixing costs: Exploring information processing in pure and mixed blocks of trials. Acta Psychologica.

[B31-behavsci-15-01481] Luk G., Bialystok E. (2013). Bilingualism is not a categorical variable: Interaction between language proficiency and usage. Journal of Cognitive Psychology.

[B32-behavsci-15-01481] Luk G., Sa E. D., Bialystok E. (2011). Is there a relation between onset age of bilingualism and enhancement of cognitive control?. Bilingualism: Language and Cognition.

[B33-behavsci-15-01481] Marian V., Blumenfeld H. K., Kaushanskaya M. (2007). The language experience and proficiency questionnaire (LEAP-Q): Assessing language profiles in bilinguals and multilinguals. Journal of Speech, Language, and Hearing Research.

[B34-behavsci-15-01481] Monsell S. (2003). Task switching. Trends in Cognitive Sciences.

[B35-behavsci-15-01481] Paap K. R., Johnson H. A., Sawi O. (2015). Bilingual advantages in executive functioning either do not exist or are restricted to very specific and undetermined circumstances. Cortex.

[B36-behavsci-15-01481] Prior A., MacWhinney B. (2010). A bilingual advantage in task switching. Bilingualism: Language and Cognition.

[B37-behavsci-15-01481] R Core Team (2023). R: A language and environment for statistical computing *(Version 4.3.0) [Computer software]*.

[B38-behavsci-15-01481] Rogers R. D., Monsell S. (1995). Costs of a predictible switch between simple cognitive tasks. Journal of Experimental Psychology: General.

[B39-behavsci-15-01481] Shannon C. E. (1948). A mathematical theory of communication. The Bell System Technical Journal.

[B40-behavsci-15-01481] Strobach T., Liepelt R., Schubert T., Kiesel A. (2012). Task switching: Effects of practice on switch and mixing costs. Psychological Research.

[B41-behavsci-15-01481] Surrain S., Luk G. (2019). Describing bilinguals: A systematic review of labels and descriptions used in the literature between 2005–2015. Bilingualism: Language and Cognition.

[B42-behavsci-15-01481] Timmermeister M., Leseman P., Wijnen F., Blom E. (2020). No bilingual benefits despite relations between language switching and task switching. Frontiers in Psychology.

[B43-behavsci-15-01481] Tiv M., Gullifer J., Feng R., Titone D. (2020). Using network science to map what Montréal bilinguals talk about across languages and communicative contexts. Journal of Neurolinguistics.

[B44-behavsci-15-01481] Tse C.-S., Altarriba J. (2014). The relationship between language proficiency and attentional control in Cantonese-English bilingual children: Evidence from Simon, Simon switching, and working memory tasks. Frontiers in Psychology.

[B45-behavsci-15-01481] Tse C.-S., Altarriba J. (2015). Local and global task switching costs in bilinguals who vary in second language proficiency. The American Journal of Psychology.

[B46-behavsci-15-01481] van den Berg F., Brouwer J., Tienkamp T. B., Verhagen J., Keijzer M. (2022). Language entropy relates to behavioral and pupil indices of executive control in young adult bilinguals. Frontiers in Psychology.

[B47-behavsci-15-01481] Vandierendonck A., Liefooghe B., Verbruggen F. (2010). Task switching: Interplay of reconfiguration and interference control. Psychological Bulletin.

[B48-behavsci-15-01481] Wang Q., Wu X., Ji Y., Yan G., Wu J. (2022). Second language proficiency modulates the dependency of bilingual language control on domain-general cognitive control. Frontiers in Psychology.

[B49-behavsci-15-01481] Ward R., Awani J. (2024). Bilingualism and flexibility in task switching: A close replication study. Studies in Second Language Acquisition.

[B50-behavsci-15-01481] Wiseheart M., Viswanathan M., Bialystok E. (2016). Flexibility in task switching by monolinguals and bilinguals. Bilingualism: Language and Cognition.

[B51-behavsci-15-01481] Wulff D. U., Kieslich P. J., Henninger F., Haslbeck J. M. (2021). Movement tracking of cognitive processes: A tutorial using mousetrap. PsyArXiv.

[B52-behavsci-15-01481] Ye W., Damian M. F. (2022). Exploring task switch costs in a color-shape decision task via a mouse tracking paradigm. Journal of Experimental Psychology: Human Perception and Performance.

[B53-behavsci-15-01481] Ye W., Damian M. F. (2023). Effects of conflict in cognitive control: Evidence from mouse tracking. Quarterly Journal of Experimental Psychology.

[B54-behavsci-15-01481] Yow W. Q., Li X. (2015). Balanced bilingualism and early age of second language acquisition as the underlying mechanisms of a bilingual executive control advantage: Why variations in bilingual experiences matter. Frontiers in Psychology.

[B55-behavsci-15-01481] Zhou B., Krott A. (2015). Data trimming procedure can eliminate bilingual cognitive advantage. Psychonomic Bulletin & Review.

[B56-behavsci-15-01481] Zhu M., Sturt P., Damian M. (2025). Language switch costs in sentence comprehension between Chinese and English: Evidence from self-paced reading. Bilingualism: Language and Cognition.

